# Role of Carvedilol in Inhibiting the Proliferation and Migration of Vascular Smooth Muscle Cells by Upregulating microRNA-145 Expression

**DOI:** 10.33549/physiolres.935564

**Published:** 2025-08-01

**Authors:** Guanrui YANG, Zhulin ZHANG, Xiurui MA, Jie CHEN, Hongtao SHI, Jinjing YANG, Qinghua HAN

**Affiliations:** 1 The First School of Clinical Medicine, Shanxi Medical University, Taiyuan, China; 2 Department of Cardiology, Shanxi Cardiovascular Hospital, Taiyuan, China; 3 Department of Cardiology, The First Hospital of Shanxi Medical University, Taiyuan, China

**Keywords:** Carvedilol, in-stent restenosis, Krüppel-like factor 4, miR-145, Vascular smooth muscle cell

## Abstract

The proliferation and migration of vascular smooth muscle cells (VSMCs) are the initial contributors to restenosis in patients undergoing percutaneous coronary intervention (PCI). MicroRNA-145 (miR-145) plays a significant role in this pathological process. Although carvedilol has been shown to inhibit VSMC proliferation and migration, the underlying mechanisms are not fully understood. The aim of our study is to examine whether carvedilol regulates the expression of miR-145 and thereby inhibits the proliferation and migrative capacity of VSMCs. VSMCs were cultured and transfected with either miR-145 mimics or miR-145 inhibitors. Cell proliferation was evaluated using the Cell Counting Kit-8 (CCK-8) and 5-ethynyl-2′-deoxyuridine (EdU) assays, while wound healing and Transwell assays were used to assess the migration capacity. Protein expression levels were quantified using western blot analysis, and additionally, a luciferase reporter assay was performed to identify the target gene of miR-145. We found that carvedilol upregulated the expression of miR-145 and decreased the expression of Krüppel-like factor 4 (KLF4). Furthermore, miR-145 inhibited VSMC proliferation and migration. KLF4 was identified as a direct target of miR-145. Importantly, the inhibition of miR-145 attenuated the suppressive effects of carvedilol on VSMCs. In summary, our results in this study demonstrate that carvedilol exerts its inhibitory effects on VSMC proliferation and migration, at least in part, through the upregulation of miR-145. These findings suggest that miR-145 may be a key mediator in the therapeutic effects of carvedilol on VSMCs.

## Introduction

In-stent restenosis (ISR) is a condition characterized by the reoccurrence of stenosis in the lumen following a successful stent implantation [1]. With millions of stents being implanted annually in patients with coronary heart disease worldwide, ISR poses a major social burden and is often referred to as the “Achilles heel” of modern percutaneous coronary intervention (PCI) [1,2]. While the exact pathological mechanisms underlying ISR are not yet fully understood, neointimal hyperplasia is widely recognized as the critical pathophysiological phenomenon involved in ISR, largely driven by the dedifferentiation of vascular smooth muscle cells (VSMCs) and their subsequent increased proliferation and migration [3].

Under normal physiological conditions, VSMCs are highly differentiated cells that are responsible for maintaining vascular tone and vasoconstriction, expressing a distinct set of unique contractile proteins such as α-smooth muscle actin and smooth muscle 22a [4]. Interestingly, VSMCs possess the ability to undergo a phenotype switch from a highly specialized contractile state to a dedifferentiation state in response to various pathophysiological stimuli such as injury and platelet-derived growth factors, among others [4]. In their dedifferentiated state, VSMCs exhibit a synthetic phenotype, characterized by increased proliferation and migration toward the intima, ultimately leading to the narrowing of the arterial lumen.

MicroRNAs (miRNAs) are small, non-coding RNA molecules that have been the focus of extensive research recently [5]. The discovery of miRNAs has revealed a new regulatory mechanism [6], positioning them as important regulators in the context of cardiovascular diseases [7]. Among these, MiR-145 is highly expressed in VSMCs and the vascular wall, where it plays a pivotal role in VSMC differentiation and modulates VSMC phenotype, thereby influencing the formation of vascular neointimal lesions [8,9]. A study by Chin et al. demonstrated that synthesized miR-145 micelles, when intravenously injected into mice, were effective in inhibiting lesion growth in atherosclerotic ApoE^−/−^ mice [10].

Carvedilol, a widely used β-blocker in clinical practice, is prescribed for patients with hypertension, angina, and heart failure [11]. Previous studies have shown that carvedilol exerts an anti-proliferative effect on VSMCs, inhibiting their migration, and has been shown to reduce intimal hyperplasia following vascular injury in animal experiments [12]. A study using a porcine ISR model demonstrated that carvedilol-coated stents could suppress neointimal formation [13]. In addition, carvedilol was found to regulate the expression of various miRNAs, including miR-125a-5p, miR-150, and miR-199a-3p, which may potentially contribute to its cardioprotective effects [14].

The aim of the present study was to investigate whether carvedilol regulates the expression of miR-145 and whether miR-145 mediates the effects of carvedilol on VSMCs. Our findings revealed that miR-145 critically mediated the inhibitory effect of carvedilol on the proliferation and migration of VSMC, in which transcriptional factor Krüppel-like factor 4 (KLF4) acts as a target for miR-145.

## Materials and Methods

### Cell experiments

Human aortic smooth muscle cells (VSMCs) were purchased from ScienCell, USA, and cultured in Smooth Muscle Cell Medium (SMCM) (ScienCell, USA) supplemented with 20 % fetal bovine serum (FBS) (BI, USA). To regulate the expression of miR-145, cells were transfected with miR-145 mimic, miR-145 inhibitor, or their respective negative controls (mimic NC, inhibitor NC), all synthesized by RiboBio (Guangzhou, China). Lipofectamine 2000 (Invitrogen, Carlsbad, CA, USA) was used for the transfection procedure.

### VSMC proliferation assays

Cell Counting Kit-8 (CCK-8) (Absin, Shanghai, China) and 5-ethynyl-2′-deoxyuridine (EdU) assays (RiboBio, Guangzhou, China) were conducted to evaluate the proliferative capacity of VSMCs. For the CCK-8 assay, cells were seeded into 96-well plates at a density of 5×10^4^ cells per well. The CCK-8 reagent was added, and 1.5 hours later, absorbance was measured at 450 nm using a microplate reader (Autobio Diagnostics, Zhengzhou, China). In the EdU assay, the VSMC proliferation rate was quantified by calculating the ratio of green-stained proliferating cells to the total number of cells observed under confocal laser scanning microscopy (Olympus, Japan). Carvedilol was administered to VSMCs at varying concentrations. After 24 hours of cultivation, miR-145 levels were measured, and cell functions were assessed. Proteins were extracted following 48 hours of co-culture.

### VSMC migration assays

The migration capacity of VSMCs was evaluated using wound healing and Transwell migration assays. In the wound healing assay, two parallel lines were scratched on the cell monolayer using a 200-μl pipette tip VSMCs were then cultured in SMCM for 16 hours. Images of the scratch boundaries were captured and analyzed to assess the extent of cell migration.

For the Transwell migration assay, VSMCs were seeded into the upper chamber of a Transwell insert without FBS, while the lower chamber was filled with SMCM containing 20 % FBS. The cells were incubated at 37°C for 24 hours. After incubation, the migrated cells in the lower chamber were fixed with 4 % paraformaldehyde (Seven, Beijing, China) and stained with crystal violet (Solarbio, Beijing, China). Images were captured using an inverted microscope (ZEISS Axiocam ERc 5s, Germany), and the average number of migrated cells was calculated.

### Luciferase reporter assay

To perform the luciferase reporter assay, 293T cells (Shanghai Cell Bank, Shanghai, China) were cultured until they reached a suitable condition for experimentation. The cells were digested, resuspended, and seeded into a 12-well plate and incubated overnight at 37°C. The cells were transfected using TGP-transfect-Mate (GenePharma, Shanghai, China). Six hours post-transfection, the medium was replaced with fresh medium, and the cells were cultured in a 37°C incubator for 48 hours.

After the incubation period, the cells were harvested for detecting luciferase activity. The medium was removed, cells were washed with phosphate-buffered saline (PBS), and 400 μL of cell lysate was added to each well and lysed for 1 hour at room temperature. Subsequently, 50 μL of cell lysate was added to an all-black 96-well plate. A volume of 50 μL of firefly luciferase reaction solution was added and mixed on a shaker plate to detect firefly luciferase activity. Finally, 50 μL of Renilla luciferase reaction solution was added and immediately mixed on the shaker, and Renilla luciferase activity was detected.

### RNA isolation and reverse transcription quantitative polymerase chain reaction (RT-qPCR)

Total RNA was extracted from VSMCs using the Trizol reagent (Invitrogen, USA). Complementary DNA (cDNA) synthesis was performed with the SweScript RT I First Strand cDNA Synthesis Kit (Servicebio, Wuhan, China). The SYBR Green Real-time PCR assay (Serbicebio, Wuhan, China) was used to detect the expression levels of miR-145 and KLF4 mRNA. The specific primers used are detailed in Table 1. U6 and GAPDH were used as internal controls. The relative expression levels were calculated using the ΔΔCq method.

### Western blot

VSMCs were lysed in RIPA buffer (Boster, Wuhan, China), and the protein concentration of the cell lysates was determined using a bicinchoninic acid (BCA) assay (Beyotime, Shanghai, China). Sodium dodecyl sulfate-polyacrylamide gel electrophoresis (SDS-PAGE) was used to separate the proteins, which were subsequently transferred onto a polyvinylidene fluoride (PVDF) membrane. After blocking with 5 % nonfat milk, the membrane was incubated overnight with rabbit polyclonal KLF4 or GAPDH primary antibodies (ProteinTech, Wuhan, China; 1:8000 dilution). Subsequently, the membrane was incubated with horseradish peroxidase-conjugated secondary antibodies (ProteinTech, Wuhan, China; 1:6000 dilution) for 2 hours at room temperature. Protein bands were visualized using an enhanced chemiluminescence kit (Boster, Wuhan, China).

### Statistical analysis

All analyses were performed in triplicate. Statistical significance was evaluated using unpaired t-tests and one-way analysis of variance (ANOVA), with a significance threshold set at *P*<0.05.

## Results

### Effects of carvedilol on the proliferation and migration of VSMCs

**Fig. 1:**
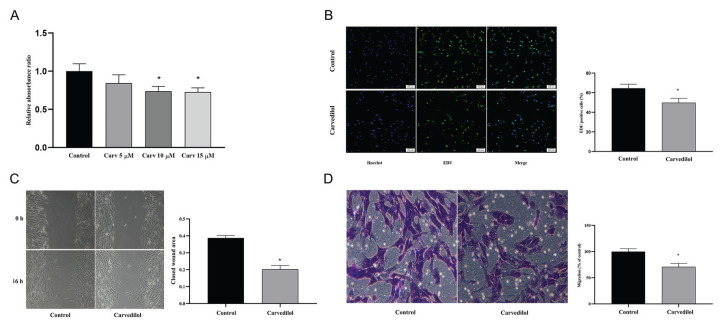
Effects of carvedilol in the proliferation and migration of VSMCs. (**A**) CCK-8 assays show the proliferation of VSMCs treated with different concentrations of carvedilol. (**B**) EdU assay illustrating VSMC proliferation in the control and carvedilol-treated groups. (**C, D**) Wound healing and Transwell migration assays of the migration capacity of VSMCs in the control and carvedilol-treated groups. * *P* < 0.05 compared with the control group. Carv, carvedilol.

VSMCs were treated with different concentrations of carvedilol to assess its impact on cellular proliferation. The CCK-8 experiment showed that VSMC proliferation capacity was significantly inhibited at carvedilol concentrations of 10 μM and 15 μM (*P*<0.05, Fig. 1A). Based on these results, 10 μM of carvedilol was chosen as the optimal concentration for subsequent experiments. The inhibitory effect of carvedilol on VSMC proliferation was further confirmed by the EdU assay (*P*<0.05, Fig. 1B). Additionally, carvedilol significantly inhibited VSMC migration in both the wound healing and Transwell assays (*P*<0.05, Figs. 1C, D).

### Effects of carvedilol on the expression of miR-145 and KLF4

**Fig. 2:**
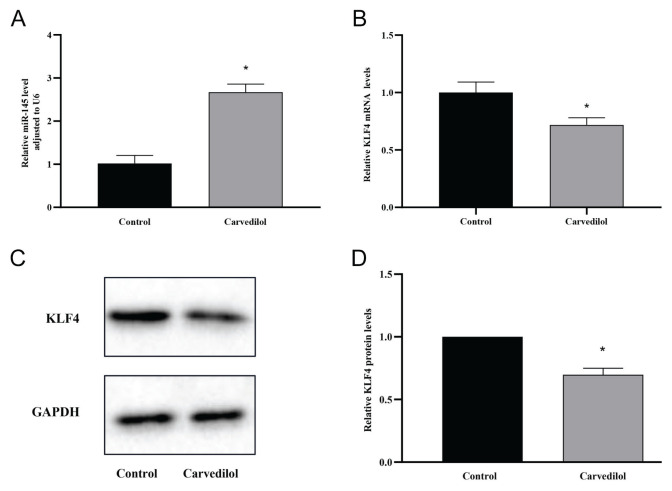
Effects of carvedilol on miR-145 and KLF4 expression. (**A**) Upregulation of miR-145 expression in VSMCs following carvedilol treatment. (**B**) Inhibition of KLF4 mRNA expression in VSMCs treated with carvedilol. (**C, D**) Decreased KLF4 protein expression in VSMCs following carvedilol treatment. * *P* < 0.05 compared with the control group. KLF4, Krüppel-like factor 4.

The expression of miR-145 was significantly upregulated in VSMCs following treatment with carvedilol (*P*<0.05, Fig. 2A). Given the close association of KLF4 with VSMCs, particularly its role in inhibiting the expression of the smooth muscle cell differentiation gene and promoting VSMC proliferation, we examined whether carvedilol has a regulatory effect on KLF4. Our observations revealed that carvedilol treatment led to a marked inhibition in the expression of KLF4 at both the mRNA and protein levels in VSMCs (*P*<0.05, Figs. 2B–D).

### Regulatory effect of miR-145 on VSMCs

Our previous experiments in this study indicated that carvedilol upregulates the expression of miR-145 and inhibits the proliferation and migration of VSMCs. To further understand whether miR-145 mediates the regulatory effects of carvedilol on VSMCs, we investigated the relationship between miR-145 and VSMC behavior.

**Fig. 3:**
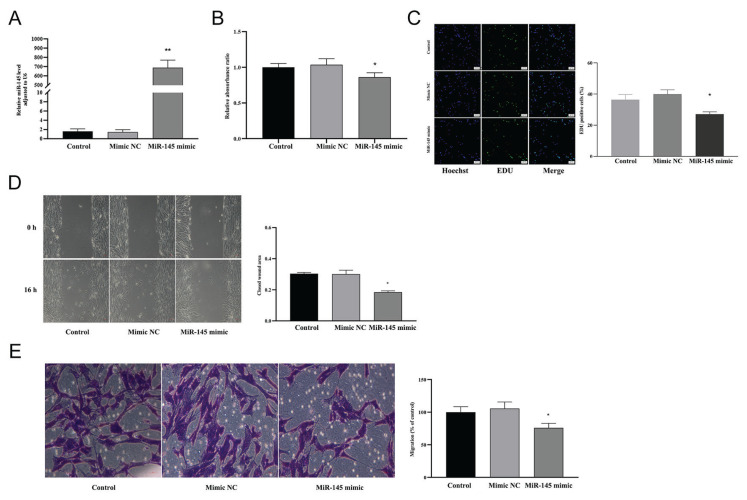
Role of miR-145 in the proliferation and migration of VSMCs. (**A**) Expression levels of miR-145 in VSMCs in the control, mimic NC, and miR-145 mimic groups. (**B, C**) CCK-8 and EdU assays demonstrating the impact of miR-145 overexpression on VSMC proliferation in the control, mimic-NC, and miR-145 mimic groups. (**D, E**) Wound healing and Transwell assays showing the effect of miR-145 overexpression on VSMC migration of VSMCs in the control, mimic NC, and miR-145 mimic groups. * *P*<0.05, ** *P*<0.01 compared with the control and mimic-NC groups.

**Fig. 4:**
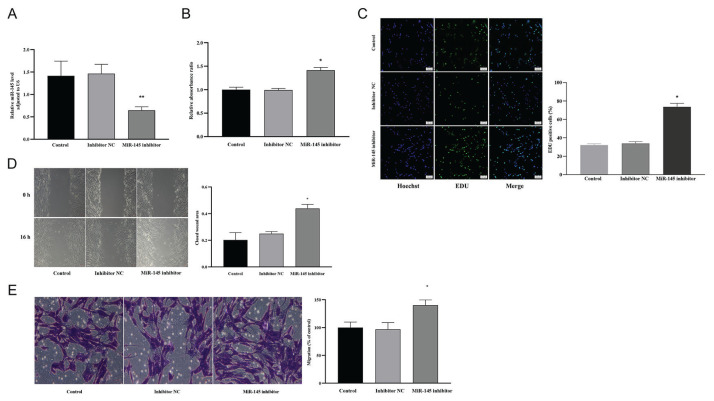
Role of miR-145 in the proliferation and migration of VSMCs. (**A**) Expression levels of miR-145 in VSMCs in the control, inhibitor NC, and miR-145 inhibitor groups. (**B, C**) CCK-8 and EdU assays illustrating the effect of miR-145 inhibition on VSMC proliferation in the control, inhibitor NC, and miR-145 inhibitor groups. (**D, E**) Wound healing and Transwell assays demonstrating the impact of miR-145 inhibition on VSMC migration in the control, inhibitor NC, and miR-145 inhibitor groups. * *P*<0.05, ** *P*<0.01 compared with the control and inhibitor NC groups.

VSMCs were successfully transfected with miR-145 mimics or inhibitors using Lipofectamine 2000, as confirmed by significant changes in miR-145 expression (*P*<0.05; Figs. 3,4A). Compared to the control group, VSMCs transfected with miR-145 mimics exhibited a significant reduction in proliferation, as assessed by both the CCK-8 and EdU assays (*P*<0.05; Figs. 3B, C). Consistent with this, the assessment of migration capacity using wound healing and Transwell assays demonstrated that VSMCs transfected with the miR-145 mimics showed significantly decreased migration compared to the control group (*P*<0.05; Figs. 3D, E). Conversely, transfection of VSMCs with miR-145 inhibitors led to a significant increase in both the proliferation and migration capacity of the cells (*P<*0.05; Figs. 4).

### KLF4 was identified as the target of miR-145

**Fig. 5:**
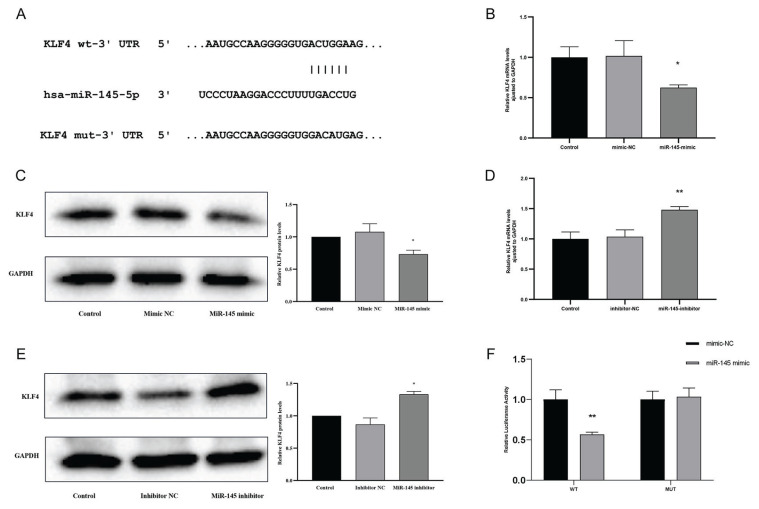
KLF4 as a target of miR-145. (**A**) Predicted binding sites of miR-145 on the KLF4 3’ UTR and corresponding mutation sites. (**B**) mRNA expression levels of KLF4 in VSMCs transfected with control, mimic NC, or miR-145 mimic. (**C**) protein levels of KLF4 in the control, mimic NC, and miR-145 mimic groups. (**D**) mRNA expression levels in the control, inhibitor NC, and miR-145 inhibitor groups. **(E**) protein levels of KLF4 in the control, inhibitor NC, and miR-145 inhibitor groups. (**F**) Luciferase activity in HEK293T cells with wild-type and mutant KLF4-UTR reporters. * *P*<0.05, ** *P*<0.01 compared with the control and mimic-NC groups.

As is known, the same miRNA can have multiple target genes. Among the various potential target genes of miR-145, in this study, we focused on KLF4, which has been shown to be closely related to VSMCs and can be downregulated by carvedilol. Extensive analysis using Target Scan 6.2 (http://www.targetscan.org/) revealed one putative binding site between miR-145 and KLF4 (Fig. 5A).

Our experiments demonstrated that the expression of KLF4 was significantly decreased in VSMCs transfected with miR-145 mimics, both at the RNA and protein levels (*P*<0.05; Figs. 5B, C). Conversely, transfection with miR-145 inhibitors resulted in a significant increase in KLF4 expression (*P*<0.05; Figs. 5D,E).

To further confirm that KLF4 is a direct target of miR-145, we conducted a luciferase reporter assay, which revealed that the transfection of miR-145 mimics into 293T cells resulted in a significant inhibition of luciferase activity, while mutation of the KLF4 3-UTR in 293T cells did not show such inhibition (*P*<0.05; Fig 5F).

These results collectively suggest that KLF4 was a direct target of miR-145 in VSMCs.

### The effects of carvedilol on VSMCs were mediated by the MiR-145-KLF4 axis

**Fig. 6:**
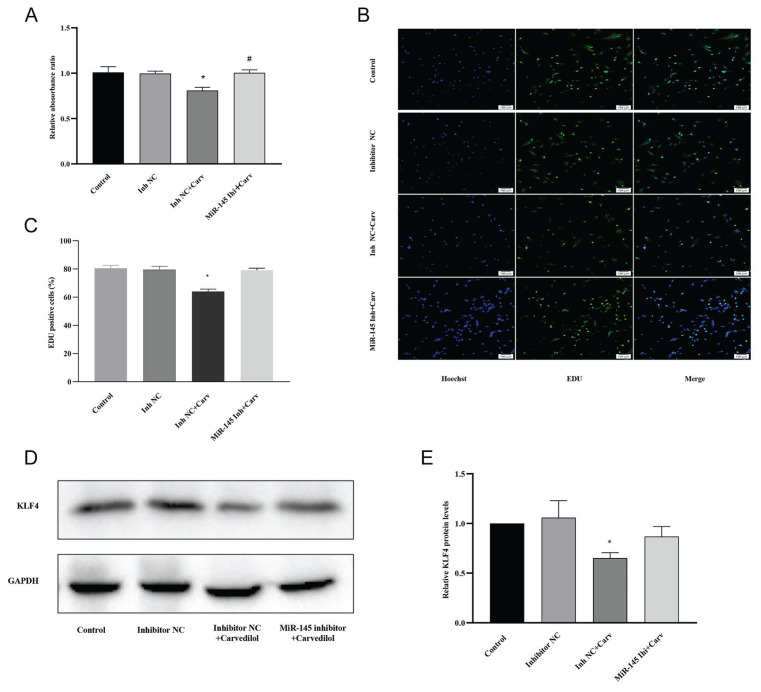
Inhibition of VSMC proliferation by carvedilol via the miR-145-KLF4 axis. (**A, B, C**) CCK-8 and EdU assays showing the effects of carvedilol on VSMC proliferation in the control, inhibitor NC, inhibitor-NC+carvedilol, and miR-145 inhibitor+carvedilol groups. (**D, E**) KLF4 expression in VSMCs in the control, inhibitor NC, inhibitor NC+carvedilol, and miR-145 inhibitor+carvedilol groups. * *P*<0.05, # *P*<0.05 compared with control and inhibitor NC groups. Inh, inhibitor; Carv, carvedilol.

To further understand whether miR-145 mediates the effects of carvedilol on VSMCs, we designed a validation experiment. The results from the CCK-8 and EDU assays showed that the inhibition of VSMC proliferation by carvedilol could be reversed by the transfection of miR-145 inhibitors (*P*<0.05; Fig. 6).

## Discussion

In this study, we demonstrated that carvedilol upregulated the expression of miR-145 while downregulating KLF4 in VSMCs, leading to inhibited proliferation and migration of these cells. The overexpression of miR-145 by miR-145 mimics resulted in reduced proliferation and migration of VSMCs, while downregulation of miR-145 in VSMCs using miR-145 inhibitors increased these capacities. KLF4 was identified as a direct target of miR-145, and importantly, the inhibitory effects of carvedilol on VSMCs were reversed by transfection with miR-145 inhibitors. Therefore, we speculate that the miR-145-KLF4 axis serves as a mediator in the effects of carvedilol on VSMCs.

PCI has been widely promoted and used worldwide as an interventional treatment for coronary heart disease. Despite advancements in stent technology and lipid-lowering therapies, ISR remains a significant clinical challenge, occurring in approximately 5 %–10 % of all coronary interventional procedures [2]. Patients with ISR tend to experience recurrent restenosis, posing difficulties for both patients and interventional cardiologists. Intimal hyperplasia caused by the excessive proliferation and migration of VSMCs has been extensively studied as a key mechanism underlying ISR.

Recently, miRNAs have gained attention as an important regulatory mechanism in various biological processes, such as VSMCs [7,15]. Among them, miR-145, encoded by a highly conserved gene cluster, is abundantly expressed in vascular walls and is selectively expressed in VSMCs, being almost undetectable in endothelial cells [3]. miR-145 is involved in several pathophysiological processes of cardiovascular diseases [16]. In a metabolic hypertensive rat model, miR-145 expression was found to be decreased, and it was involved in the maintenance of vasoconstriction and regulation of vascular remodeling [17]. In another study, a significant decrease in plasma miR-145 expression levels was noted in individuals who were pre-diabetic as well as patients with diabetes [18]. Among patients with in-stent restenosis, plasma levels of miR-143 and miR-145 were decreased significantly, suggesting that these miRNAs could serve as potential biomarkers for in-stent restenosis [19].

Importantly, research studies in rat carotid artery balloon-injury models have demonstrated that the upregulation of miR-145 can significantly reduce neointima formation [8, 20]. miR-145 is involved in the phenotypic switching of VSMCs and intimal hyperplasia following vascular injury through various mechanisms [20, 21]. Consistent with these earlier findings, our study revealed that the proliferation and migration capabilities of VSMCs were inhibited by the upregulation of miR-145. Conversely, the downregulation of miR-145 using miR-145 inhibitors resulted in the opposite effects, highlighting the regulatory importance of miR-145 in VSMC behavior.

MiR-145 is known to target several genes, including Elk-1, myocardin, JAM-A, and CaMKIIδ [9, 22, 23]. Among these targets of miR-145 is KLF4, a transcriptional regulator extensively studied for its roles in VSMC differentiation and phenotype switching [22–27]. Previous studies have demonstrated its role in suppressing cell proliferation, while recent research highlights its critical and complex regulatory function during phenotypic switching of VSMCs. KLF4 downregulates the expression of contractile genes (e.g., ACTA2, MYH11,) by inhibiting the synergistic interaction between MYOCD (myocardin) and SRF (serum response factor), thereby weakening the contractile phenotype of VSMCs. Additionally, KLF4 promotes phenotypic transitions of VSMCs into macrophage-like cells (*via* cholesterol uptake and foam cell formation), osteogenic-like cells, fibroblast-like cells, and even adipocyte-like cells [28]. Our research further confirmed that the upregulation of miR-145 leads to a significant decrease in KLF4 expression, while the inhibition of miR-145 results in increased KLF4 levels. The presence of a potential binding site in the miR-145 sequence for KLF4 was further confirmed using a dual luciferase reporter assay, reinforcing the direct regulatory relationship between miR-145 and KLF4. These findings suggest that carvedilol may exerts its inhibitory effects on VSMC proliferation and migration through the miR-145/KLF4 signaling axis. Certainly, it would be beneficial to incorporate experiments involving the knockout of KLF4 to validate its mediating role in the regulatory process of carvedilol and miR-145.

Carvedilol, a third-generation β-receptor blocker, is extensively used in clinical settings. It has a unique pharmacological profile, including high affinity for both β and α receptor blockers, calcium antagonism, and notable anti-free radical and antioxidant properties. These characteristics enable carvedilol to inhibit smooth muscle cell proliferation while also preventing myocardial cell apoptosis [12, 29–31]. Among the three β-blockers—carvedilol, metoprolol succinate, and bisoprolol—proven beneficial in clinical trials, carvedilol is particularly recommended by various guidelines due to its broad therapeutic effects. For instance, in a clinical study on patients receiving regular hemodialysis, carvedilol was found to reduce the incidence of arteriovenous graft failure [32].

Understanding the mechanisms underlying carvedilol is of utmost importance for optimizing its clinical use. Park et al. reported that carvedilol inhibits the activation of ERK 1/2 and p38, indicating its involvement in the MAPK signaling pathway [29]. This is particularly interesting given that miR-145 is also implicated in MAPK signaling in several studies [33, 34]. Additionally, carvedilol has been shown to regulate the expression of various miRNAs across different studies, further suggesting a complex regulatory role in cellular signaling and function [14, 35, 36].

However, the relationship between carvedilol and miR-145 expression, as well as the potential role of miR-145 in mediating the effects of carvedilol on VSMCs, remain poorly understood. There is evidence from our study that carvedilol significantly upregulates miR-145 expression in VSMCs. Notably, the inhibitions of miR-145 could reverse the suppressive effects of carvedilol on VSMCs, indicating that miR-145 plays a critical role in mediating these effects. Furthermore, we confirmed that miR-145 downregulates KLF4 expression, establishing KLF-4 as a direct target of miR-145. The miR-145-KLF4 axis, therefore, was found to serve as a mediator in the regulatory influence of carvedilol on VSMCs. This discovery enhances the current understanding of the underlying mechanisms involved in the cardiac and vascular protective effects of carvedilol and also opens up new avenues to further explore the interaction between pharmacological agents and miRNAs.

It is important to acknowledge certain limitations in this study. This study was restricted to in vitro cell experiments to observe and evaluate the role of miR-145 in mediating the effects of carvedilol on VSMCs. Consequently, this mechanism requires further validation through animal experiments and clinical trials to fully establish its relevance and applicability.

The phenotypic switching of VSMCs is a key mechanism contributing to in-stent restenosis, involving a complex regulatory network of miRNAs and proteins. Further research is required to explore this regulatory mechanism in detail. Clinically, many patients with in-stent restenosis also have coexisting hypertension or heart failure—conditions for which β-blockers are strongly indicated. Given that carvedilol has been used safely and effectively worldwide for over 30 years, we recommend that carvedilol be prioritized for these patients to enhance cardiac prognosis and improve coronary outcomes.

## Figures and Tables

**Fig. 7:**
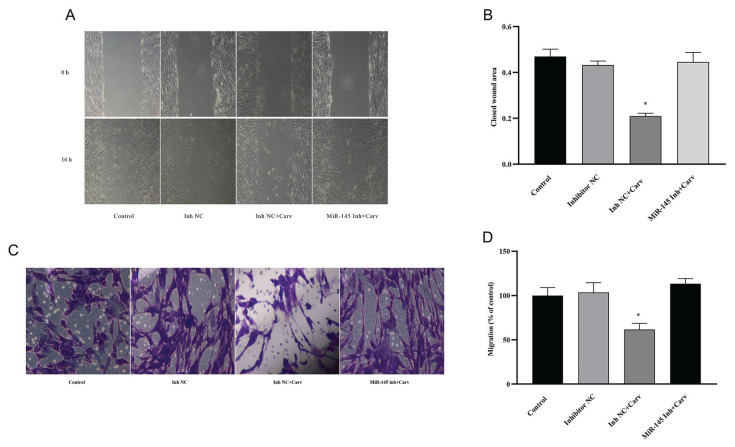
Inhibition of the migration of VSMCs by carvedilol via miR-145. (**A, B, C, D**) Wound healing and Transwell invasion assays of VSMCs in the control, inhibitor NC, inhibitor-NC+carvedilol, and miR-145 inhibitor+carvedilol groups. * *P*<0.05, # *P*<0.05 compared with control and inhibitor-NC groups. Inh, inhibitor; Carv, carvedilol.

**Table 1:** Primer sequences of KLF4, GAPDH, mir-145-5p, and U6.

Gene symbol	Sequence (5′-3′)	Product size,bp	GeneBank accession no.
KLF4	F: 5′-CCCACATGAAGCGACTTCCC-3′ R: 5′-CAGGTCCAGGAGATCGTTGAA-3′	170	NM_004235
GAPDH	F: 5′-GGAGCGAGATCCCTCCAAAAT-3′ R: 5′-GGCTGTTGTCATACTTCTCATGG-3′	197	NM_001256799
miR-145	RT:5′-CTCAACTGGTGTCGTGGAGTCGGCAATTCAGTTGAGAGGGATTC-3′ F: 5′-ACACTCCAGCTGGGGTCCAGTTTTCCCAGGA-3′ R: 5′-TGGTGTCGTGGAGTCG-3′	67	NR_029686
U6	F: 5′-CTCGCTTCGGCAGCACA-3′ R: 5′-AACGCTTCACGAATTTGCGT-3′	94	NR_029686

KLF4, Krüppel-like factor 4; mir-145, microRNA-145.

## Abbreviations

VSMCs: vascular smooth muscle cells
miR-145: microRNA-145
ISR: in-stent restenosis
PCI: percutaneous coronary intervention
KLF4: Krüppel-like factor 4
